# 5-Cyclo­hexyl-2-(4-methyl­phen­yl)-3-methyl­sulfinyl-1-benzofuran

**DOI:** 10.1107/S1600536812037294

**Published:** 2012-09-05

**Authors:** Hong Dae Choi, Pil Ja Seo, Uk Lee

**Affiliations:** a Department of Chemistry, Dongeui University, San 24 Kaya-dong Busanjin-gu, Busan 614-714, Republic of Korea; b Department of Chemistry, Pukyong National University, 599-1 Daeyeon 3-dong Nam-gu, Busan 608-737, Republic of Korea

## Abstract

In the title compound, C_22_H_24_O_2_S, the cyclo­hexyl ring adopts a chair conformation. In the crystal, mol­ecules are linked by weak C—H⋯O and C—H⋯π inter­actions. In the methyl­sulfinyl group, the methyl group and S atom are disordered over two sets of sites, with site-occupancy factors of 0.58 (3) and 0.42 (3). In the ring of the 4-methyl­phenyl group, the four C atoms are disordered over two sets of sites, with site-occupancy factors of 0.858 (5) and 0.142 (5).

## Related literature

For background information and the crystal structures of related compounds, see: Choi *et al.* (2011*a* ▶,*b* ▶).
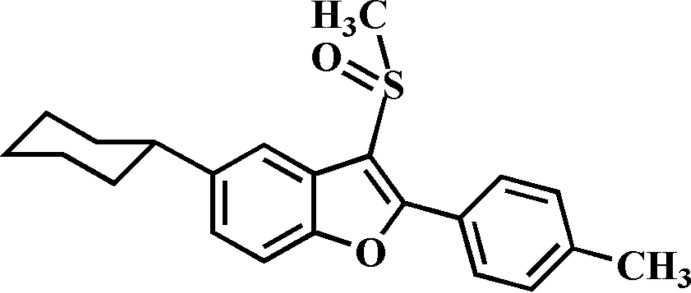


## Experimental

### 

#### Crystal data

C_22_H_24_O_2_S
*M* *_r_* = 352.47
Monoclinic, 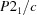

*a* = 16.4392 (5) Å
*b* = 7.2726 (2) Å
*c* = 15.8433 (5) Å
β = 108.652 (1)°
*V* = 1794.67 (9) Å^3^
*Z* = 4
Mo *K*α radiation
μ = 0.19 mm^−1^
*T* = 173 K
0.40 × 0.33 × 0.29 mm

#### Data collection

Bruker SMART APEXII CCD diffractometer
Absorption correction: multi-scan (*SADABS*; Bruker, 2009 ▶) *T*_min_ = 0.671, *T*_max_ = 0.746
16617 measured reflections
4447 independent reflections
3204 reflections with *I* > 2σ(*I*)
*R*_int_ = 0.037

#### Refinement

*R*[*F* ^2^ > 2σ(*F* ^2^)] = 0.064
*wR*(*F* ^2^) = 0.204
*S* = 1.06
4447 reflections
280 parameters
83 restraints
H-atom parameters constrained
Δρ_max_ = 0.84 e Å^−3^
Δρ_min_ = −0.68 e Å^−3^

### 

Data collection: *APEX2* (Bruker, 2009 ▶); cell refinement: *SAINT* (Bruker, 2009 ▶); data reduction: *SAINT*; program(s) used to solve structure: *SHELXS97* (Sheldrick, 2008 ▶); program(s) used to refine structure: *SHELXL97* (Sheldrick, 2008 ▶); molecular graphics: *ORTEP-3* (Farrugia, 2012 ▶) and *DIAMOND* (Brandenburg, 1998 ▶); software used to prepare material for publication: *SHELXL97*.

## Supplementary Material

Crystal structure: contains datablock(s) global, I. DOI: 10.1107/S1600536812037294/xu5615sup1.cif

Structure factors: contains datablock(s) I. DOI: 10.1107/S1600536812037294/xu5615Isup2.hkl

Supplementary material file. DOI: 10.1107/S1600536812037294/xu5615Isup3.cml

Additional supplementary materials:  crystallographic information; 3D view; checkCIF report

## Figures and Tables

**Table 1:** Hydrogen-bond geometry (Å, °) *Cg* is the centroid of the C2–C7 benzene ring.

*D*—H⋯*A*	*D*—H	H⋯*A*	*D*⋯*A*	*D*—H⋯*A*
C6—H6⋯O2^i^	0.95	2.60	3.405 (3)	143
C21—H21*A*⋯*Cg* ^ii^	0.98	2.89	3.619 (3)	137
C21—H21*C*⋯*Cg* ^iii^	0.98	2.97	3.665 (3)	137

Symmetry codes: (i) 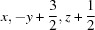; (ii) 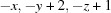; (iii) 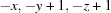.

**Fig. 1.**
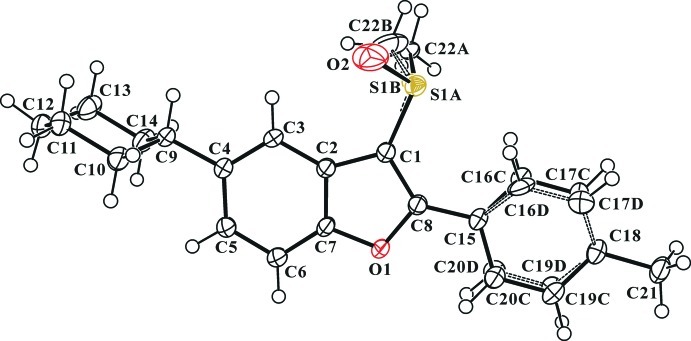
The molecular structure of the title compound with the atom numbering scheme. Displacement ellipsoids are drawn at the 50% probability level. H atoms are presented as small spheres of arbitrary radius. The C22 and S1 atoms of the methylsulfinyl group is disordered over two positions with site occupancy factors, from refinement of 0.58 (3) (part A) and 0.42 (3) (part B). In the phenyl ring of the 4-methylphenyl group, the C16/C17/C19/C20 atoms are disordered over two positions with site-occupancy factors, from refinement of 0.858 (5) (part C) and 0.142 (5) (part D).

**Fig. 2.**
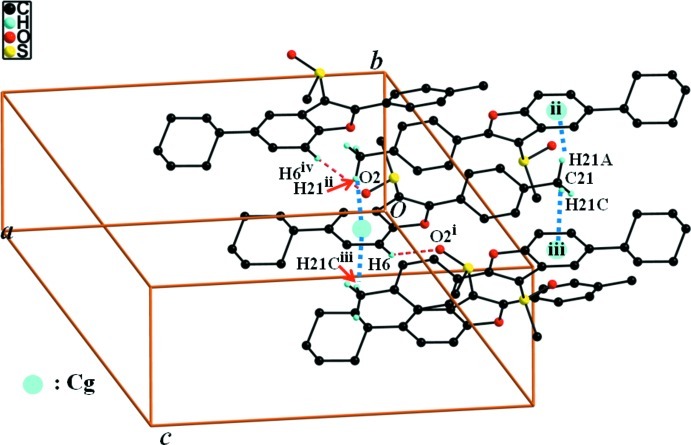
A view of the C–H···O and C–H···π interactions (dotted lines) in the crystal structure of the title compound. H atoms non-participating in hydrogen-bonding and disordered part B and D atoms were omitted for clarity. [Symmetry codes: (i) x, - y + 3/2, z + 1/2; (ii) - x, - y + 2, - z + 1; (iii) - x, - y + 1 ,- z + 1; (iv) x, - y + 3/2, z - 1/2.]

## supplementary crystallographic information

### Comment

As a part of our ongoing study of 5-cyclohexyl-3-methylsulfinyl-1-benzofuran derivatives containing phenyl (Choi *et al.*, 2011*a*) and 4-fluorophenyl (Choi *et al.*, 2011*b*) substituents in 2-position, we report herein the crystal structure of the title compound. In the title molecule (Fig. 1), the benzofuran unit is essentially planar, with a mean deviation of 0.006 (2) Å from the least-squares plane defined by the nine constituent atoms. The cyclohexyl ring has a chair conformation. In the methylsulfinyl group, the C22 and S1 atoms are disordered over two positions with site-occupancy factors, from refinement of 0.58 (3) (part A) and 0.42 (3) (part B). In the phenyl ring of the 4-methylphenyl group, the four C atoms (C16/C17/C19/C20) are disordered over two positions with site-occupancy factors, from refinement of 0.858 (5) (part C) and 0.142 (5) (part D). In the crystal structure (Fig. 2), molecules are connected by weak C–H···O and C–H···π interactions (Table 1, Cg is the centroid of the C2-C7 benzene ring).

### Experimental

3-Chloroperoxybenzoic acid (77%, 224 mg, 1.0 mmol) was added in small portions to a stirred solution of 5-cyclohexyl-2-(4-methylphenyl)-3-methylsulfanyl-1-benzofuran (302 mg, 0.9 mmol) in dichloromethane (30 mL) at 273 K. After being stirred at room temperature for 4h, the mixture was washed with saturated sodium bicarbonate solution and the organic layer was separated, dried over magnesium sulfate, filtered and concentrated at reduced pressure. The residue was purified by column chromatography (hexane-ethyl acetate, 1:1 v/v) to afford the title compound as a colorless solid [yield 74%, m.p. 442-443 K; *R*_f_ = 0.63 (hexane-ethyl acetate, 1:1 v/v)]. Single crystals suitable for X-ray diffraction were prepared by slow evaporation of a solution of the title compound in acetone at room temperature.

### Refinement

All H atoms were geometrically positioned and refined using a riding model, with C–H = 0.95 Å for aryl, 1.00 Å for methine, 0.99 Å for methylene and 0.98 Å for methyl H atoms, respectively. Uiso(H) = 1.2Ueq(C) for aryl, methine and methylene, and 1.5Ueq(C) for methyl H atoms. The positions of methyl, methylene and methine hydrogens were optimized rotationally. The C22 and S1 atoms of the methylsulfinyl group is disordered over two positions with site-occupancy factors, from refinement of of 0.58 (3) (part A) and 0.42 (3) (part B). The distance of equivalent S–C and S–O pairs were restrained to 1.790 (3) and 1.500 (3) Å using command DFIX, and displacement ellipsoids of C22 and S1 set were restrained to 0.01 using SHELXL command ISOR, respectively. In the phenyl ring of the 4-methylphenyl group, the C16/C17/C19/C20 atoms are disordered over two positions with site-occupancy factors, from refinement of 0.858 (5) (part C) and 0.142 (5) (part D). The distance of equivalent C-C pairs were restrained to 1.400 (3) Å using command DFIX, and displacement ellipsoids of C16/C17/C19/C20 sets were restrained to 0.01 using command ISOR.

### Crystal data

**Table:**

C_22_H_24_O_2_S	*F*(000) = 752
*M**_r_* = 352.47	*D*_x_ = 1.305 Mg m^−^^3^
Monoclinic, *P*2_1_/*c*	Mo *K*α radiation, λ = 0.71073 Å
Hall symbol: -P 2ybc	Cell parameters from 5036 reflections
*a* = 16.4392 (5) Å	θ = 2.6–28.3°
*b* = 7.2726 (2) Å	µ = 0.19 mm^−^^1^
*c* = 15.8433 (5) Å	*T* = 173 K
β = 108.652 (1)°	Block, colourless
*V* = 1794.67 (9) Å^3^	0.40 × 0.33 × 0.29 mm
*Z* = 4	

### Data collection

**Table:**

Bruker SMART APEXII CCD diffractometer	4447 independent reflections
Radiation source: rotating anode	3204 reflections with *I* > 2σ(*I*)
Graphite multilayer monochromator	*R*_int_ = 0.037
Detector resolution: 10.0 pixels mm^-1^	θ_max_ = 28.3°, θ_min_ = 2.6°
φ and ω scans	*h* = −21→21
Absorption correction: multi-scan (*SADABS*; Bruker, 2009)	*k* = −9→7
*T*_min_ = 0.671, *T*_max_ = 0.746	*l* = −20→21
16617 measured reflections	

### Refinement

**Table:**

Refinement on *F*^2^	Primary atom site location: structure-invariant direct methods
Least-squares matrix: full	Secondary atom site location: difference Fourier map
*R*[*F*^2^ > 2σ(*F*^2^)] = 0.064	Hydrogen site location: difference Fourier map
*wR*(*F*^2^) = 0.204	H-atom parameters constrained
*S* = 1.06	*w* = 1/[σ^2^(*F*_o_^2^) + (0.1164*P*)^2^ + 0.6896*P*] where *P* = (*F*_o_^2^ + 2*F*_c_^2^)/3
4447 reflections	(Δ/σ)_max_ < 0.001
280 parameters	Δρ_max_ = 0.84 e Å^−^^3^
83 restraints	Δρ_min_ = −0.68 e Å^−^^3^

### Special details

**Table:**

Geometry. All esds (except the esd in the dihedral angle between two l.s. planes) are estimated using the full covariance matrix. The cell esds are taken into account individually in the estimation of esds in distances, angles and torsion angles; correlations between esds in cell parameters are only used when they are defined by crystal symmetry. An approximate (isotropic) treatment of cell esds is used for estimating esds involving l.s. planes.
Refinement. Refinement of F^2^ against ALL reflections. The weighted R-factor wR and goodness of fit S are based on F^2^, conventional R-factors R are based on F, with F set to zero for negative F^2^. The threshold expression of F^2^ > 2sigma(F^2^) is used only for calculating R-factors(gt) etc. and is not relevant to the choice of reflections for refinement. R-factors based on F^2^ are statistically about twice as large as those based on F, and R- factors based on ALL data will be even larger.

### Fractional atomic coordinates and isotropic or equivalent isotropic displacement parameters (Å^2^)

**Table:**

	*x*	*y*	*z*	*U*_iso_*/*U*_eq_	Occ. (<1)
S1A	0.1071 (3)	0.7252 (6)	0.3361 (4)	0.0252 (8)	0.58 (3)
S1B	0.1117 (5)	0.7405 (10)	0.3352 (6)	0.0397 (18)	0.42 (3)
O1	0.12467 (10)	0.75238 (19)	0.59015 (11)	0.0241 (4)	
O2	0.17368 (13)	0.6013 (3)	0.32049 (13)	0.0552 (6)	
C1	0.13693 (13)	0.7551 (3)	0.45074 (15)	0.0221 (5)	
C2	0.22173 (14)	0.7527 (3)	0.51491 (15)	0.0212 (5)	
C3	0.30555 (14)	0.7503 (3)	0.51048 (15)	0.0218 (5)	
H3	0.3151	0.7486	0.4544	0.026*	
C4	0.37431 (14)	0.7506 (3)	0.58871 (15)	0.0215 (5)	
C5	0.35942 (15)	0.7514 (3)	0.67138 (16)	0.0258 (5)	
H5	0.4072	0.7515	0.7245	0.031*	
C6	0.27707 (15)	0.7520 (3)	0.67783 (16)	0.0268 (5)	
H6	0.2672	0.7526	0.7337	0.032*	
C7	0.21044 (14)	0.7518 (3)	0.59833 (15)	0.0225 (5)	
C8	0.07917 (13)	0.7542 (3)	0.50148 (15)	0.0216 (5)	
C9	0.46566 (14)	0.7488 (3)	0.58568 (15)	0.0227 (5)	
H9	0.4622	0.7484	0.5215	0.027*	
C10	0.51523 (14)	0.5758 (4)	0.62860 (16)	0.0328 (6)	
H10A	0.5195	0.5715	0.6923	0.039*	
H10B	0.4835	0.4652	0.5992	0.039*	
C11	0.60521 (15)	0.5740 (4)	0.62039 (17)	0.0385 (6)	
H11A	0.6367	0.4648	0.6516	0.046*	
H11B	0.6009	0.5644	0.5568	0.046*	
C12	0.65480 (17)	0.7463 (4)	0.65976 (19)	0.0453 (8)	
H12A	0.6662	0.7467	0.7250	0.054*	
H12B	0.7108	0.7453	0.6488	0.054*	
C13	0.60627 (15)	0.9196 (4)	0.62008 (17)	0.0389 (7)	
H13A	0.6020	0.9283	0.5565	0.047*	
H13B	0.6383	1.0283	0.6512	0.047*	
C14	0.51605 (14)	0.9202 (4)	0.62825 (16)	0.0319 (6)	
H14A	0.5204	0.9252	0.6920	0.038*	
H14B	0.4849	1.0313	0.5988	0.038*	
C15	−0.01307 (13)	0.7544 (3)	0.47842 (13)	0.0220 (5)	
C18	−0.19280 (14)	0.7538 (3)	0.44006 (14)	0.0258 (5)	
C16C	−0.06670 (13)	0.8143 (4)	0.39572 (16)	0.0267 (7)	0.858 (5)
H16C	−0.0427	0.8566	0.3521	0.032*	0.858 (5)
C17C	−0.15553 (14)	0.8122 (4)	0.37691 (17)	0.0286 (7)	0.858 (5)
H17C	−0.1914	0.8514	0.3200	0.034*	0.858 (5)
C19C	−0.13836 (14)	0.7002 (4)	0.52330 (16)	0.0257 (6)	0.858 (5)
H19C	−0.1623	0.6630	0.5678	0.031*	0.858 (5)
C20C	−0.04973 (15)	0.6999 (4)	0.54265 (18)	0.0271 (7)	0.858 (5)
H20C	−0.0139	0.6623	0.5999	0.033*	0.858 (5)
C16D	−0.0668 (7)	0.685 (3)	0.3974 (6)	0.031 (4)	0.142 (5)
H16D	−0.0424	0.6353	0.3555	0.038*	0.142 (5)
C17D	−0.1562 (7)	0.687 (3)	0.3774 (8)	0.034 (4)	0.142 (5)
H17D	−0.1920	0.6438	0.3213	0.041*	0.142 (5)
C19D	−0.1381 (7)	0.795 (3)	0.5255 (5)	0.024 (4)*	0.142 (5)
H19D	−0.1620	0.8212	0.5715	0.029*	0.142 (5)
C20D	−0.0490 (8)	0.800 (3)	0.5447 (7)	0.030 (4)	0.142 (5)
H20D	−0.0130	0.8337	0.6025	0.035*	0.142 (5)
C21	−0.28860 (15)	0.7517 (3)	0.4204 (2)	0.0347 (6)	
H21A	−0.3136	0.8620	0.3865	0.052*	
H21B	−0.3018	0.7496	0.4765	0.052*	
H21C	−0.3127	0.6420	0.3854	0.052*	
C22A	0.1276 (5)	0.9500 (7)	0.3058 (4)	0.0223 (16)	0.58 (3)
H22A	0.1869	0.9844	0.3390	0.033*	0.58 (3)
H22B	0.0881	1.0365	0.3198	0.033*	0.58 (3)
H22C	0.1194	0.9539	0.2417	0.033*	0.58 (3)
C22B	0.1599 (16)	0.9450 (17)	0.3132 (10)	0.069 (4)	0.42 (3)
H22D	0.2224	0.9357	0.3393	0.103*	0.42 (3)
H22E	0.1393	1.0500	0.3394	0.103*	0.42 (3)
H22F	0.1446	0.9626	0.2487	0.103*	0.42 (3)

### Atomic displacement parameters (Å^2^)

**Table:**

	*U*^11^	*U*^22^	*U*^33^	*U*^12^	*U*^13^	*U*^23^
S1A	0.0228 (13)	0.0305 (14)	0.0237 (15)	−0.0006 (8)	0.0095 (10)	−0.0053 (9)
S1B	0.0250 (19)	0.077 (4)	0.017 (2)	−0.0061 (18)	0.0069 (14)	0.003 (2)
O1	0.0168 (7)	0.0356 (10)	0.0215 (8)	0.0001 (6)	0.0086 (6)	−0.0012 (6)
O2	0.0683 (14)	0.0619 (14)	0.0433 (11)	0.0228 (11)	0.0290 (10)	0.0001 (10)
C1	0.0187 (10)	0.0254 (11)	0.0227 (11)	0.0004 (8)	0.0074 (8)	−0.0009 (8)
C2	0.0191 (10)	0.0236 (11)	0.0215 (10)	0.0002 (8)	0.0073 (8)	−0.0009 (8)
C3	0.0201 (10)	0.0266 (12)	0.0216 (10)	0.0008 (8)	0.0106 (8)	0.0001 (8)
C4	0.0183 (10)	0.0231 (11)	0.0239 (11)	0.0001 (8)	0.0080 (8)	−0.0001 (8)
C5	0.0207 (10)	0.0346 (13)	0.0213 (11)	0.0001 (9)	0.0057 (8)	−0.0015 (9)
C6	0.0220 (11)	0.0382 (14)	0.0215 (11)	−0.0002 (9)	0.0087 (9)	−0.0008 (9)
C7	0.0167 (10)	0.0284 (12)	0.0245 (11)	−0.0001 (8)	0.0096 (8)	−0.0011 (8)
C8	0.0192 (10)	0.0238 (11)	0.0222 (11)	−0.0006 (8)	0.0071 (8)	−0.0013 (8)
C9	0.0176 (10)	0.0295 (12)	0.0226 (11)	0.0006 (8)	0.0086 (8)	0.0003 (8)
C10	0.0270 (11)	0.0382 (14)	0.0364 (13)	0.0080 (10)	0.0144 (10)	0.0104 (11)
C11	0.0281 (12)	0.0531 (17)	0.0378 (13)	0.0172 (12)	0.0154 (10)	0.0153 (13)
C12	0.0200 (11)	0.087 (3)	0.0273 (13)	0.0037 (12)	0.0054 (10)	−0.0006 (13)
C13	0.0252 (11)	0.0562 (18)	0.0375 (13)	−0.0143 (12)	0.0130 (10)	−0.0143 (13)
C14	0.0242 (11)	0.0401 (14)	0.0339 (12)	−0.0059 (10)	0.0127 (9)	−0.0091 (11)
C15	0.0179 (10)	0.0220 (11)	0.0275 (12)	−0.0007 (8)	0.0095 (9)	−0.0028 (8)
C18	0.0195 (10)	0.0250 (12)	0.0342 (13)	−0.0004 (8)	0.0107 (9)	−0.0049 (9)
C16C	0.0212 (12)	0.0342 (17)	0.0264 (13)	−0.0013 (11)	0.0101 (10)	−0.0003 (12)
C17C	0.0196 (12)	0.0377 (18)	0.0272 (13)	−0.0018 (11)	0.0056 (10)	−0.0010 (12)
C19C	0.0235 (13)	0.0254 (16)	0.0329 (15)	−0.0007 (11)	0.0156 (11)	−0.0009 (11)
C20C	0.0220 (13)	0.0310 (17)	0.0295 (14)	0.0006 (12)	0.0100 (10)	0.0022 (13)
C16D	0.036 (7)	0.041 (9)	0.028 (7)	−0.003 (6)	0.024 (6)	−0.002 (6)
C17D	0.035 (7)	0.037 (9)	0.030 (7)	−0.001 (7)	0.011 (6)	0.005 (7)
C20D	0.026 (7)	0.036 (8)	0.027 (7)	−0.003 (6)	0.010 (6)	0.002 (7)
C21	0.0201 (11)	0.0448 (16)	0.0410 (14)	−0.0021 (10)	0.0123 (10)	−0.0026 (11)
C22A	0.021 (3)	0.025 (3)	0.022 (2)	−0.0064 (18)	0.010 (2)	0.0050 (16)
C22B	0.074 (8)	0.095 (7)	0.053 (5)	−0.046 (6)	0.041 (6)	−0.013 (5)

### Geometric parameters (Å, º)

**Table:**

S1A—O2	1.499 (2)	C13—H13A	0.9900
S1A—C1	1.736 (6)	C13—H13B	0.9900
S1A—C22A	1.767 (3)	C14—H14A	0.9900
S1B—O2	1.507 (3)	C14—H14B	0.9900
S1B—C1	1.745 (9)	C15—C16C	1.395 (2)
S1B—C22B	1.772 (3)	C15—C20C	1.396 (2)
O1—C8	1.364 (3)	C15—C20D	1.400 (3)
O1—C7	1.374 (2)	C15—C16D	1.400 (3)
C1—C8	1.427 (2)	C18—C19C	1.392 (2)
C1—C2	1.439 (3)	C18—C17C	1.396 (2)
C2—C7	1.392 (3)	C18—C19D	1.398 (3)
C2—C3	1.402 (3)	C18—C17D	1.401 (3)
C3—C4	1.385 (3)	C18—C21	1.505 (3)
C3—H3	0.9500	C16C—C17C	1.394 (2)
C4—C5	1.407 (3)	C16C—H16C	0.9500
C4—C9	1.518 (3)	C17C—H17C	0.9500
C5—C6	1.390 (3)	C19C—C20C	1.390 (2)
C5—H5	0.9500	C19C—H19C	0.9500
C6—C7	1.380 (3)	C20C—H20C	0.9500
C6—H6	0.9500	C16D—C17D	1.401 (3)
C8—C15	1.442 (3)	C16D—H16D	0.9500
C9—C14	1.529 (3)	C17D—H17D	0.9500
C9—C10	1.535 (3)	C19D—C20D	1.398 (3)
C9—H9	1.0000	C19D—H19D	0.9500
C10—C11	1.526 (3)	C20D—H20D	0.9500
C10—H10A	0.9900	C21—H21A	0.9800
C10—H10B	0.9900	C21—H21B	0.9800
C11—C12	1.517 (4)	C21—H21C	0.9800
C11—H11A	0.9900	C22A—H22A	0.9800
C11—H11B	0.9900	C22A—H22B	0.9800
C12—C13	1.516 (4)	C22A—H22C	0.9800
C12—H12A	0.9900	C22B—H22D	0.9800
C12—H12B	0.9900	C22B—H22E	0.9800
C13—C14	1.529 (3)	C22B—H22F	0.9800
			
O2—S1A—C1	105.7 (3)	C14—C13—H13B	109.4
O2—S1A—C22A	107.3 (3)	H13A—C13—H13B	108.0
C1—S1A—C22A	98.9 (3)	C9—C14—C13	111.32 (19)
O2—S1B—C1	104.9 (4)	C9—C14—H14A	109.4
O2—S1B—C22B	99.6 (7)	C13—C14—H14A	109.4
C1—S1B—C22B	100.6 (6)	C9—C14—H14B	109.4
C8—O1—C7	107.78 (16)	C13—C14—H14B	109.4
C8—C1—C2	105.71 (19)	H14A—C14—H14B	108.0
C8—C1—S1A	124.9 (2)	C16C—C15—C20C	118.9 (2)
C2—C1—S1A	128.6 (2)	C16C—C15—C20D	109.9 (7)
C8—C1—S1B	127.8 (3)	C20C—C15—C16D	105.8 (6)
C2—C1—S1B	126.3 (3)	C20D—C15—C16D	118.7 (8)
C7—C2—C3	118.6 (2)	C16C—C15—C8	122.42 (19)
C7—C2—C1	106.12 (19)	C20C—C15—C8	118.64 (19)
C3—C2—C1	135.3 (2)	C20D—C15—C8	118.2 (6)
C4—C3—C2	119.3 (2)	C16D—C15—C8	122.4 (5)
C4—C3—H3	120.4	C19C—C18—C17C	117.9 (2)
C2—C3—H3	120.4	C17C—C18—C19D	109.5 (6)
C3—C4—C5	119.9 (2)	C19C—C18—C17D	106.4 (7)
C3—C4—C9	120.3 (2)	C19D—C18—C17D	118.2 (8)
C5—C4—C9	119.9 (2)	C19C—C18—C21	120.4 (2)
C6—C5—C4	122.1 (2)	C17C—C18—C21	121.7 (2)
C6—C5—H5	118.9	C19D—C18—C21	120.6 (6)
C4—C5—H5	118.9	C17D—C18—C21	120.5 (5)
C7—C6—C5	116.1 (2)	C17C—C16C—C15	120.1 (2)
C7—C6—H6	121.9	C17C—C16C—H16C	119.9
C5—C6—H6	121.9	C15—C16C—H16C	119.9
O1—C7—C6	125.3 (2)	C16C—C17C—C18	121.3 (2)
O1—C7—C2	110.78 (19)	C16C—C17C—H17C	119.3
C6—C7—C2	124.0 (2)	C18—C17C—H17C	119.3
O1—C8—C1	109.61 (18)	C20C—C19C—C18	121.4 (2)
O1—C8—C15	116.54 (17)	C20C—C19C—H19C	119.3
C1—C8—C15	133.8 (2)	C18—C19C—H19C	119.3
C4—C9—C14	112.41 (17)	C19C—C20C—C15	120.4 (2)
C4—C9—C10	112.55 (17)	C19C—C20C—H20C	119.8
C14—C9—C10	109.7 (2)	C15—C20C—H20C	119.8
C4—C9—H9	107.3	C15—C16D—C17D	120.7 (10)
C14—C9—H9	107.3	C15—C16D—H16D	119.6
C10—C9—H9	107.3	C17D—C16D—H16D	119.6
C11—C10—C9	111.17 (19)	C16D—C17D—C18	120.1 (10)
C11—C10—H10A	109.4	C16D—C17D—H17D	119.9
C9—C10—H10A	109.4	C18—C17D—H17D	119.9
C11—C10—H10B	109.4	C18—C19D—C20D	121.4 (11)
C9—C10—H10B	109.4	C18—C19D—H19D	119.3
H10A—C10—H10B	108.0	C20D—C19D—H19D	119.3
C12—C11—C10	111.5 (2)	C19D—C20D—C15	119.7 (11)
C12—C11—H11A	109.3	C19D—C20D—H20D	120.2
C10—C11—H11A	109.3	C15—C20D—H20D	120.2
C12—C11—H11B	109.3	C18—C21—H21A	109.5
C10—C11—H11B	109.3	C18—C21—H21B	109.5
H11A—C11—H11B	108.0	H21A—C21—H21B	109.5
C13—C12—C11	112.0 (2)	C18—C21—H21C	109.5
C13—C12—H12A	109.2	H21A—C21—H21C	109.5
C11—C12—H12A	109.2	H21B—C21—H21C	109.5
C13—C12—H12B	109.2	S1B—C22B—H22D	109.5
C11—C12—H12B	109.2	S1B—C22B—H22E	109.5
H12A—C12—H12B	107.9	H22D—C22B—H22E	109.5
C12—C13—C14	111.3 (2)	S1B—C22B—H22F	109.5
C12—C13—H13A	109.4	H22D—C22B—H22F	109.5
C14—C13—H13A	109.4	H22E—C22B—H22F	109.5
C12—C13—H13B	109.4		
			
C1—S1A—O2—S1B	−92 (6)	C9—C10—C11—C12	55.7 (3)
C22A—S1A—O2—S1B	13 (6)	C10—C11—C12—C13	−54.2 (3)
C1—S1B—O2—S1A	82 (6)	C11—C12—C13—C14	54.1 (3)
C22B—S1B—O2—S1A	−174 (6)	C4—C9—C14—C13	−177.1 (2)
O2—S1A—C1—C8	−138.2 (3)	C10—C9—C14—C13	56.9 (3)
C22A—S1A—C1—C8	110.9 (3)	C12—C13—C14—C9	−55.8 (3)
O2—S1A—C1—C2	29.8 (5)	O1—C8—C15—C16C	158.9 (2)
C22A—S1A—C1—C2	−81.1 (4)	C1—C8—C15—C16C	−21.2 (4)
O2—S1A—C1—S1B	91 (3)	O1—C8—C15—C20C	−18.4 (3)
C22A—S1A—C1—S1B	−20 (2)	C1—C8—C15—C20C	161.5 (2)
O2—S1B—C1—C8	−134.1 (4)	O1—C8—C15—C20D	16.1 (10)
C22B—S1B—C1—C8	122.9 (8)	C1—C8—C15—C20D	−164.0 (10)
O2—S1B—C1—C2	39.5 (7)	O1—C8—C15—C16D	−154.0 (10)
C22B—S1B—C1—C2	−63.6 (9)	C1—C8—C15—C16D	25.9 (10)
O2—S1B—C1—S1A	−82 (2)	C20C—C15—C16C—C17C	−2.5 (4)
C22B—S1B—C1—S1A	175 (3)	C20D—C15—C16C—C17C	−34.3 (9)
C8—C1—C2—C7	−0.4 (2)	C16D—C15—C16C—C17C	77.1 (8)
S1A—C1—C2—C7	−170.1 (3)	C8—C15—C16C—C17C	−179.8 (2)
S1B—C1—C2—C7	−175.1 (3)	C15—C16C—C17C—C18	1.1 (4)
C8—C1—C2—C3	179.2 (2)	C19C—C18—C17C—C16C	1.0 (4)
S1A—C1—C2—C3	9.5 (4)	C19D—C18—C17C—C16C	31.2 (8)
S1B—C1—C2—C3	4.5 (5)	C17D—C18—C17C—C16C	−79.8 (9)
C7—C2—C3—C4	−1.2 (3)	C21—C18—C17C—C16C	179.8 (2)
C1—C2—C3—C4	179.3 (2)	C17C—C18—C19C—C20C	−1.6 (4)
C2—C3—C4—C5	0.6 (3)	C19D—C18—C19C—C20C	−81.4 (12)
C2—C3—C4—C9	−179.76 (18)	C17D—C18—C19C—C20C	37.6 (8)
C3—C4—C5—C6	0.0 (3)	C21—C18—C19C—C20C	179.5 (2)
C9—C4—C5—C6	−179.66 (19)	C18—C19C—C20C—C15	0.2 (4)
C4—C5—C6—C7	0.0 (3)	C16C—C15—C20C—C19C	1.9 (4)
C8—O1—C7—C6	179.2 (2)	C20D—C15—C20C—C19C	81.8 (12)
C8—O1—C7—C2	−0.2 (2)	C16D—C15—C20C—C19C	−38.6 (8)
C5—C6—C7—O1	−179.97 (19)	C8—C15—C20C—C19C	179.3 (2)
C5—C6—C7—C2	−0.6 (3)	C16C—C15—C16D—C17D	−76.9 (17)
C3—C2—C7—O1	−179.35 (17)	C20C—C15—C16D—C17D	39.6 (18)
C1—C2—C7—O1	0.3 (2)	C20D—C15—C16D—C17D	10 (2)
C3—C2—C7—C6	1.2 (3)	C8—C15—C16D—C17D	179.9 (13)
C1—C2—C7—C6	−179.1 (2)	C15—C16D—C17D—C18	−2 (3)
C7—O1—C8—C1	−0.1 (2)	C19C—C18—C17D—C16D	−36.2 (18)
C7—O1—C8—C15	179.79 (16)	C17C—C18—C17D—C16D	78.3 (17)
C2—C1—C8—O1	0.3 (2)	C19D—C18—C17D—C16D	−8 (2)
S1A—C1—C8—O1	170.5 (2)	C21—C18—C17D—C16D	−178.1 (13)
S1B—C1—C8—O1	174.9 (3)	C19C—C18—C19D—C20D	82.3 (18)
C2—C1—C8—C15	−179.6 (2)	C17C—C18—C19D—C20D	−30.3 (18)
S1A—C1—C8—C15	−9.3 (4)	C17D—C18—C19D—C20D	10 (2)
S1B—C1—C8—C15	−5.0 (4)	C21—C18—C19D—C20D	−179.3 (13)
C3—C4—C9—C14	118.1 (2)	C18—C19D—C20D—C15	−3 (3)
C5—C4—C9—C14	−62.3 (3)	C16C—C15—C20D—C19D	35.0 (19)
C3—C4—C9—C10	−117.5 (2)	C20C—C15—C20D—C19D	−78.6 (17)
C5—C4—C9—C10	62.2 (3)	C16D—C15—C20D—C19D	−7 (2)
C4—C9—C10—C11	177.2 (2)	C8—C15—C20D—C19D	−177.8 (13)
C14—C9—C10—C11	−56.8 (3)		

### Hydrogen-bond geometry (Å, º)

Cg is the centroid of the C2–C7 benzene ring.

**Table:**

*D*—H···*A*	*D*—H	H···*A*	*D*···*A*	*D*—H···*A*
C6—H6···O2^i^	0.95	2.60	3.405 (3)	143
C21—H21*A*···*Cg*^ii^	0.98	2.89	3.619 (3)	137
C21—H21*C*···*Cg*^iii^	0.98	2.97	3.665 (3)	137

Symmetry codes: (i) *x*, −*y*+3/2, *z*+1/2; (ii) −*x*, −*y*+2, −*z*+1; (iii) −*x*, −*y*+1, −*z*+1.

## References

[bb1] Brandenburg, K. (1998). *DIAMOND* Crystal Impact GbR, Bonn, Germany.

[bb2] Bruker (2009). *APEX2*, *SADABS* and *SAINT* Bruker AXS Inc., Madison, Wisconsin, USA.

[bb3] Choi, H. D., Seo, P. J., Son, B. W. & Lee, U. (2011*a*). *Acta Cryst.* E**67**, o281. 10.1107/S1600536810054358 PMC3051680 21522973

[bb4] Choi, H. D., Seo, P. J., Son, B. W. & Lee, U. (2011*b*). *Acta Cryst.* E**67**, o470. 10.1107/S1600536811002297 PMC3051523 21523129

[bb5] Farrugia, L. J. (2012). *J. Appl. Cryst.* **45**, 849–854.

[bb6] Sheldrick, G. M. (2008). *Acta Cryst.* A**64**, 112–122. 10.1107/S0108767307043930 18156677

